# P-744. Admission Avoidance: A Protocol for Using Dalbavancin in the Emergency Department for Acute Bacterial Skin and Skin Structure Infections

**DOI:** 10.1093/ofid/ofae631.940

**Published:** 2025-01-29

**Authors:** Katherine Weller, Christopher M Bland, Bruce M Jones

**Affiliations:** University of Georgia College of Pharmacy, Savannah, Georgia; University of Georgia College of Pharmacy, Savannah, Georgia; St. Joseph's/Candler Health System, Savannah, GA

## Abstract

**Background:**

Dalbavancin is a glycopeptide with a 14.4 day terminal half-life that has been used previously in our institution to facilitate early discharge for acute bacterial skin and skin structure infections (ABSSSI). We have expanded this program with a pharmacist-driven protocol for use in the emergency department (ED) to avoid admission in ABSSSI for selected patients. The goal of this study was to evaluate the protocol for dalbavancin use in the ED for patient selection and financial impact.

Dalbavancin Emergency Department Protocol

**Figure:**
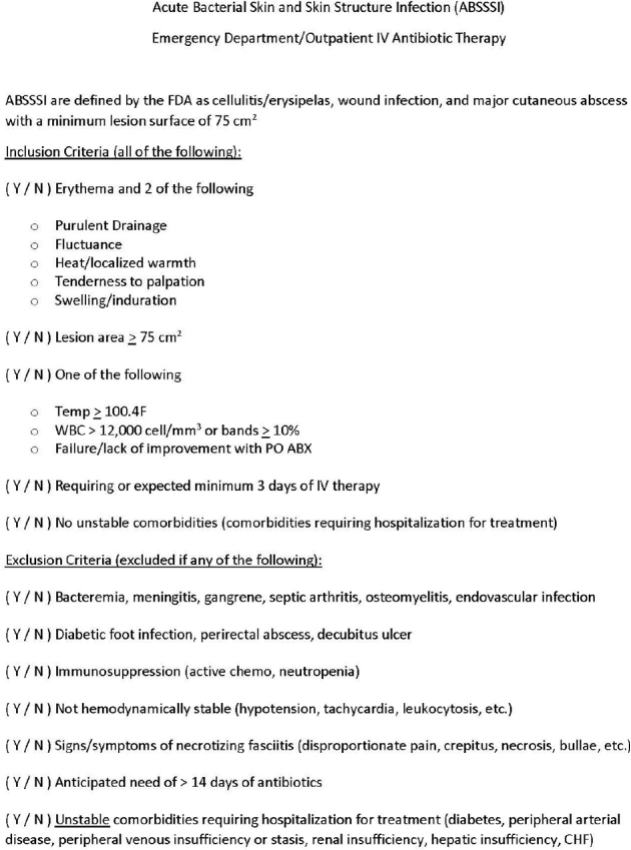

Inclusion/Exclusion criteria for assessment to receive dalbavancin

**Methods:**

This was a retrospective, multi-center, study performed in a community health system from June 2022 through March 2024 of patients who presented to the ED with ABSSSI, evaluated by pharmacist and physician, and discharged directly after receiving dalbavancin. Inclusion and exclusion criteria were based on the patient's symptoms, type of infection, and comorbidities (Figure 1). Data collected included clinical signs/symptoms of infection, previous antibiotic use, history of substance abuse or methicillin-resistant *Staphylococcus aureus* (MRSA), renal function, and readmissions. Direct costs and reimbursement, as well as if they received patient assistance, were also tracked. The primary objective was to evaluate adherence to the protocol for use of dalbavancin in the ED. The secondary objective was to analyze the financial impact to the health system.

Table 1 & Table 2

**Figure:**
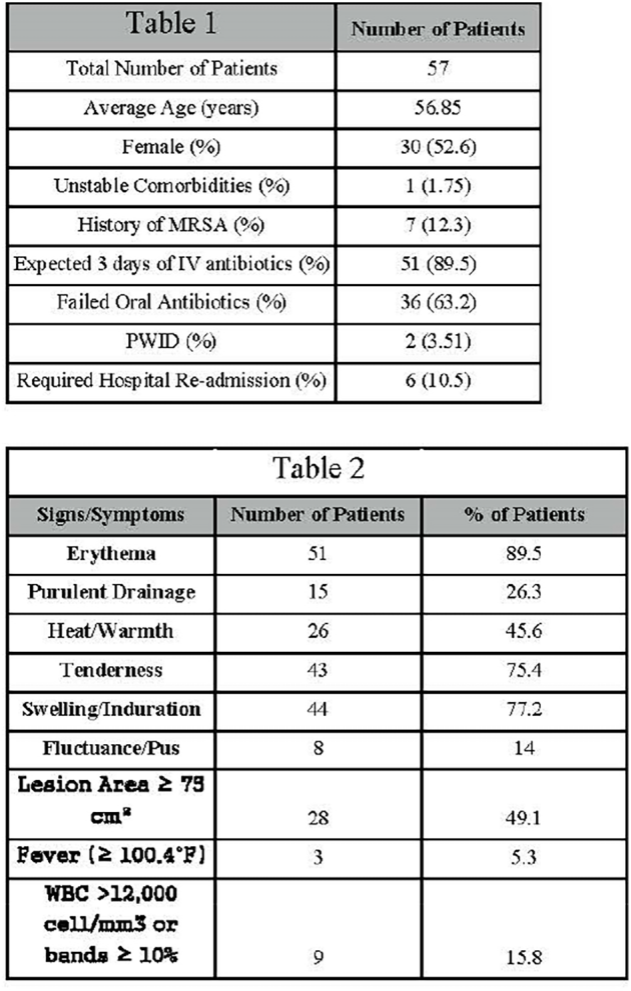

Patient Demographics & Clinical Signs/Symptoms

**Results:**

A total of 57 patients met inclusion/exclusion criteria and were evaluated. Patient demographics associated with adherence to protocol are summarized in Table 1. A majority of patients had failed oral therapy and were expected to be admitted for > 3 days. Six patients (10.5%) were re-admitted within 30 days, similar to national averages. Only 2 patients self-reported as persons who inject drugs (PWID). Table 2 summarizes clinical signs/symptoms associated with diagnosis of ABSSSI for the included population. Most patients met multiple diagnostic criteria, with erythema, tenderness, & swelling all reported >75%. For the secondary objective, patients as part of the ED visit had ∼$3800 average reimbursement per patient not including direct and indirect costs of care.

**Conclusion:**

Pharmacists can help identify and facilitate appropriate patients to receive dalbavancin within a structured protocol to help prevent admissions for ABSSSI.

**Disclosures:**

**Christopher M. Bland, PharmD, FCCP, FIDSA, BCPS**, Merck: Honoraria|Nestle/Seres: Honoraria|Shionogi: Advisor/Consultant|Shionogi: Honoraria **Bruce M. Jones, Pharm.D., FIDSA, BCPS**, AbbVie: Advisor/Consultant|AbbVie: Honoraria|Ferring: Honoraria|Innoviva: Honoraria|Paratek: Honoraria

